# Neural evidence for persistent attentional bias to threats in patients with social anxiety disorder

**DOI:** 10.1093/scan/nsy101

**Published:** 2018-11-15

**Authors:** So-Yeon Kim, Jung Eun Shin, Yoonji Irene Lee, Haena Kim, Hang Joon Jo, Soo-Hee Choi

**Affiliations:** 1 Department of Psychology, Duksung Women’s University, Seoul, Republic of Korea; 2 Department of Psychiatry, Seoul National University Hospital, Seoul, Republic of Korea; 3 Department of Psychological and Brain Sciences, Texas A&M University, College Station, TX, USA; 4 Department of Neurology, Mayo Clinic, Rochester, MN, USA; 5 Department of Neurosurgery, Mayo Clinic, Rochester, MN, USA; 6 Department of Psychiatry, Seoul National University College of Medicine and Institute of Human Behavioral Medicine, SNU-MRC, Seoul 03080, Republic of Korea

**Keywords:** social anxiety disorder, attentional bias, fMRI, inferior frontal gyrus, temporo-parietal junction

## Abstract

Does the biased attention toward social threats dwells on or disappears in patients with social anxiety disorder (SAD)? We investigated the neural mechanism of attentional bias in terms of attentional capture and holding in SAD. A total of 31 SAD patients and 30 healthy controls performed a continuous performance task detecting the orientation of a red letter ‘T’ while angry or neutral face distractors appeared or disappeared at the center of the screen. Behaviorally, typical attentional capture effects were found in response to abruptly appearing distractors in both groups. The patient group showed significant attentional dwelling effects in response to the angry face distractor only. Patients showed increased neural activity in the amygdala, insula/inferior frontal gyrus (IFG) and temporo-parietal junction (TPJ) compared with those of controls for the abruptly appearing angry distractor. Patients also maintained increased activities in brain regions related to attentional reorienting to distractor, namely the TPJ and IFG in line with their behavioral results of attentional holding effects. Our results indicate that patients with SAD showed prolonged attentional bias to task-irrelevant social threats. The underlying mechanism of prolonged attentional bias in SAD was indicated with amygdala hyperactivity and continued activity of the bottom-up attention network including the TPJ and IFG.

## Introduction

Social anxiety disorder (SAD) is characterized by fear or anxiety about social situations and emotional hyper-reactivity to social threats (Etkin and Wager, 2007; Stein and Stein, 2008; Freitas Ferrari *et al.*, 2010). Similar to individuals with other anxiety disorders, who exhibit an attentional bias for threatening stimuli (Bar-Haim *et al.*, 2007), patients with SAD also show distorted attention to negatively salient stimuli such as a negative evaluation from others (Schultz and Heimberg, 2008; Choi *et al.*, 2016). This attentional bias to social threats plays a central role in provoking and maintaining social fear in patients with SAD (Clark and Wells, 1995; Rapee and Heimberg, 1997) and modifying biased attention often reduces symptoms of SAD (Heeren *et al.*, 2016a, b). However, it is not well understood how long attentional bias persists in response to a social threat in patients with SAD. Additionally, uncertainty exists regarding whether attention dwells on or moves away from environmental threats in patients with SAD because mixed results have been reported in various experimental circumstances (Gilboa-Schechtman *et al.*, 1999; Chen *et al.*, 2002; Mansell *et al.*, 2003; Spector *et al.*, 2003). Recently, McTeague *et al.* (2018) reported a sustained bias for hypervigilance or avoidance depending on the severity of functional impairments in patients with SAD using visual-evoked potentials.

The ability to maintain focus and avoid distraction from goal-irrelevant distractors is critical when performing many tasks. However, abrupt onset of a new task-irrelevant distractor automatically captures attention although healthy adults can easily disengage from the distractor when all the distractors are from the same category, such as faces (Kim and Hopfinger, 2010; Parks *et al.*, 2014). Previous studies reported that attentional shifting to the distractor and reorienting to a target were associated with neural activation in the intraparietal sulcus (IPS) and temporo-parietal junction (TPJ) in healthy individuals (Hopfinger *et al.*, 2000; Corbetta and Shulman, 2002; Hahn *et al.*, 2006). It is well known that anxiety impairs efficient functioning of the goal-directed attentional system (Eysenck *et al.*, 2007), and task-irrelevant social stimuli often capture attention, impairing SAD patients’ social functions. Moreover, recent studies have added understanding of relation between anxiety and attention in SAD by discovering moderators of initial attentional bias in social anxiety (Judah *et al.*, 2013; Taylor *et al.*, 2016; McTeague *et al.*, 2018). That is, higher degree to self-focus may reduce attentional bias to threat in SAD and highly anxious individuals (Judah *et al.*, 2013). A recent study also reported non-linear and distinct patterns of initial attentional capture to threat in SAD based on symptom severity, demonstrating that the most severe SAD patients showed avoidant patterns to social threats (McTeague *et al.*, 2018). Despite increasing evidence on initial attentional bias to threat in SAD both at neural and behavioral levels, to our knowledge, neural mechanisms of persistent attentional bias to threats in SAD have not been reported. Thus, here we examined whether neural correlates of attentional bias in SAD would be found in the similar brain areas as those in healthy controls, such as in IPS and TPJ. Furthermore, we investigated persistent attentional bias to threats and its neural correlates in patients with SAD in the limbic regions, including the amygdala and insula (Freitas Ferrari *et al.*, 2010; Brühl *et al.*, 2014).

We investigated behavioral and neural mechanisms of attentional bias in terms of attentional capture and attentional holding in patients with SAD. A modified continuous performance task with face distractors (either neutral or angry faces) was used to separate the initial capture of attention from the subsequent holding of attention (Kim and Hopfinger, 2010; Parks *et al.*, 2014). Specifically, participants were asked to detect targets while ignoring central distractors, and behavioral attentional bias to threat was defined as impairment in target detection performance: either attentional capture [i.e. reaction time (RT) increase on targets with onset of emotional distractors] or attentional holding (i.e. dwelling of RT increase on targets with emotional distractor). To examine the attentional bias at the neural level, emotion- and attention-related regions were selected as candidate brain regions. First, the limbic regions, such as amygdala and insula, as well as distractor-related areas, namely fusiform face area (FFA), were selected as areas of interest associated with emotion and attentional bias. Further, brain regions associated with attentional orientation were predefined. Specifically, the IPS is a core region of the dorsal attention network, which embodies the top-down control mechanism and is involved in sending top-down signals that bias processing of the appropriate stimulus in the sensory cortex (Bundesen, 1990). The TPJ and inferior frontal gyrus (IFG) are core regions of the ventral attentional network, which manifests the stimulus-driven control mechanism, and are activated when attention is reoriented to a behaviorally relevant object (Corbetta *et al.*, 2000).

We hypothesized that initial attentional capture to distractors that impairs task performance at the time of each distractor’s onset would occur under both distractor conditions of angry and neutral faces in both patients with SAD and healthy controls. Based on previous findings (Parks *et al.*, 2014), we also predicted that attentional holding effects (AHEs) would not occur in both distractor conditions in healthy controls, while the extended attentional holding impairing performance beyond the distractor onset would be evident in a particularly angry face condition in patients with SAD. We also predicted that the underlying neural mechanism of pathological attentional bias would be found in attentional networks along with the affective network.

## Materials and methods

### Participants and measurements

Participants (31 patients with SAD and 30 healthy controls) were recruited from the psychiatric outpatient clinic at Seoul National University Hospital and the community through an advertisement.

All participants received intensive clinical interviews with a psychiatrist (SAD group) and/or psychologist (both SAD and control groups) after screened using online self-reported questionnaires, including the Liebowitz Social Anxiety Scale (LSAS; Liebowitz, 1987), Social Interaction Anxiety Scale (SIAS) and Social Phobia Scale (SPS; Mattick and Clarke, 1998), brief version of the Fear of Negative Evaluation Scale (B-FNE; Leary, 1983) and Beck Depression Inventory (BDI; Beck *et al.*, 1961). The inclusion criteria were LSAS ≥ 30, SIAS ≥ 34 and/or SPS ≥ 24 for patients; SIAS < 34, SPS < 24, B-FNE < 48 and BDI < 21 for controls; and right-handed and ≥12 years of education for both groups. The exclusion criteria for both groups included any history of medical, neurological or psychiatric illness (other than SAD and related secondary depressive disorder). After screening, all participants underwent structured interview with the Mini International Neuropsychiatric Interview (Lecrubier *et al.*, 1997; Sheehan *et al.*, 1997). Through this interview, we excluded control participants when they had psychiatric disorders including social anxiety and depressive disorders. Patients were diagnosed as SAD when they met full criteria for SAD according to the criteria of the *Diagnostic and Statistical Manual of Mental Disorders* (fifth edition, Washington) through an additional intensive clinical interview with a psychiatrist (Soo-Hee Choi). Four participants with SAD were excluded during this clinical interview because they did not meet the full criteria for SAD. General anxiety symptoms were assessed using the Hamilton Anxiety Scale (HAS; Hamilton, 1959). Four patients with SAD also diagnosed with comorbid depressive disorders. Seven patients were taking routine medications mainly with serotonergic antidepressants. One patient was prescribed benzodiazepines for as-needed medication; however, he/she did not take on the day of scanning.

As presented in Table 1, demographic variables including age, sex and education were not significantly different between the two groups. Patients showed higher scores on the HAS and BDI as well as higher social anxiety scores than controls. Study protocol was approved by the institutional review board at Seoul National University Hospital and was conducted in accordance with the Declaration of Helsinki. Written informed consent was obtained from all participants.

**Table 1:** Demographic and descriptive characteristics of the participants

**Variable**	**SAD (*N* = 31)**		**Controls (*N* = 30)**		***χ*** ^***2***^ **or *t***
	***N***	**%**	***N***	**%**	
Male	15	48.4	16	53.3	0.149
	**Mean**	**s.d.**	**Mean**	**s.d.**	
Age, in years	25.4	3.0	25.3	3.0	0.113
Educational level, in years	15.4	2.3	15.6	1.4	−0.447
LSAS, 0–144	76.1	25.8	17.8	7.5	12.094^*^
SIAS, 0–80	52.7	14.4	12.0	6.5	14.251^*^
SPS, 0–80	39.1	19.1	4.5	3.8	9.882^*^
B-FNE, 12–60	47.6	9.7	25.5	6.8	10.376^*^
HAS^a^, 0–56	28.3	8.7	6.6	5.1	11.341^*^
BDI, 0–63	17.9	10.7	3.4	5.0	6.785^*^

^a^Four patients with SAD were missing.

^*^ *P* < 0.001

### Continuous performance task design

**Fig. 1:**
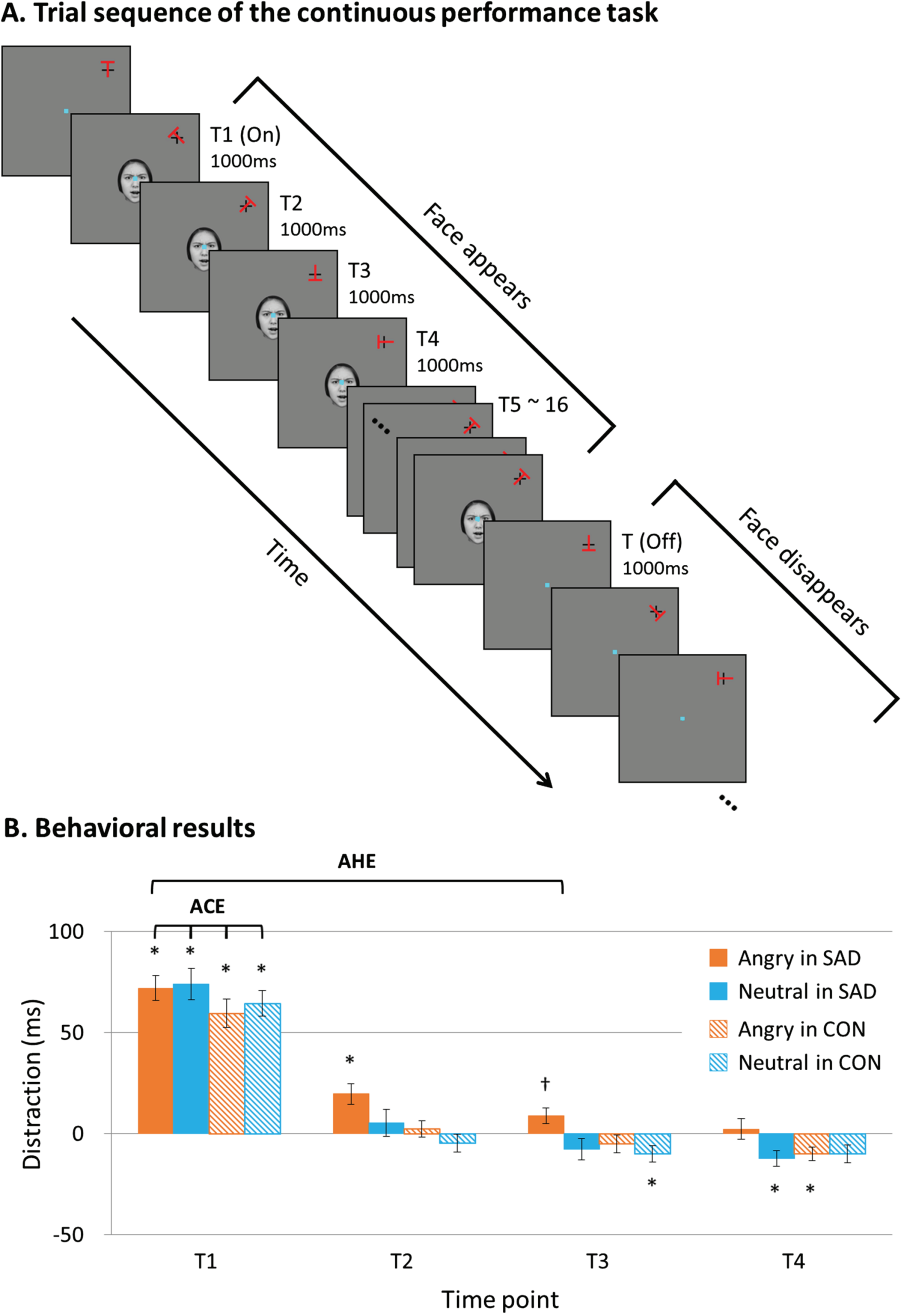
Example of the task and the behavioral results. **(A)** Each frame is presented for 1 s, and orientation of the target letter ‘T’ changed between each frame. The duration between successive distractor onsets and offsets was equally drawn from the durations of 5, 6, 8 and 16 s; the example depicts 5 s. While maintaining central fixation, participants discriminated the orientation of each target. **(B)** Both patient and control groups demonstrated the ACE upon appearance of an angry or neutral face distractor. The patient group exhibited the AHE on the angry face distractor. Distraction refers to reaction times relative to respective T-baseline (all Ts other than T1–T4). CON refers to controls. *Significant RT differences *vs* T-baseline at significance after B–H correction for multiple comparisons. ^†^Trend level significance.

Functional magnetic resonance imaging (fMRI) was conducted while participants performed the continuous performance task (Figure 1A) as modified from previous studies (Kim and Hopfinger, 2010; Parks *et al.*, 2014). Participants were asked to focus on a fixation dot in the middle of the screen. A target (red ‘T’) was presented in the upper right corner of the screen (5.88° × 5.88°) overlapping a black crosshair. The orientation of the target was randomly changed every second requiring target responses every second but presented at all times to discard further attentional dwell time caused by a newly presented target. Participants were instructed to press the left button of the button box if the target direction was horizontal or vertical to the crosshair, that is, 0°, 90°, 180°, or 270°, and the right button for the diagonally presented target, that is, 45°, 135°, 225°, or 315°. The distractors comprised grayscale images of emotional faces with either a neutral or an angry emotion. Eight angry and neutral faces were selected from the Korean Facial Expressions of Emotion database based on inter-rater reliability (i.e. ≥80% of reliability; Park *et al.*, 2011). As in the previous study (Parks *et al.*, 2014), the distractors were centrally presented overlapping the fixation dot during the course of the orientation discrimination task. The location of distractors was purposely selected to maximize distraction effects during a covert attention task (Parks *et al.*, 2014). Additionally, the peripheral location of the target allows us to catch neural correlates of attentional orienting and reorienting as participants were instructed to pay attention to the peripheral target while ignoring faces presented at the center of the screen. The distractors were centrally presented overlapping the fixation dot during the course of the orientation discrimination task. Each face distractors appeared and disappeared for 5, 6, 8 or 16 s, which resulted in various interstimulus intervals (ISIs) between distractor onset and offset, in order to allow jittering for our fMRI design. To distribute the number of trials equally across different conditions and different ISIs, we created six runs of the continuous performance task. Each run consisted of eight angry and eight neutral conditions and lasted 280 s, resulting in 280 target decisions per run.

Prior to the fMRI session, a short video clip of a practice run was shown to familiarize the participants with the stimuli and the procedure.

Before the task, a functional localizer run was conducted to identify the volumes of interest (VOIs) in the visual cortex for the peripheral target and emotion-related regions for the emotional distractor. See the Supplementary data for more information on the functional localizer run and the protocols of imaging acquisition and preprocessing.

### Imaging acquisition and preprocessing

Brain images were acquired using a 3.0 Tesla MR scanner (Magnetom TrioTrim; Siemens Medical Solutions, Erlangen, Germany). Functional images were obtained using an echo planar imaging sequence (matrix size = 64 × 64, number of slices = 34, slices thickness = 3.4 mm, spatial resolution = 3.4 × 3.4 × 3.4 mm^3^, TE = 30 ms, TR = 2 s, field of view = 220 × 220 mm, flip angle = 80 degrees). In all functional runs, data from the first three volumes were discarded to allow for stabilization of magnetic fields. High-resolution anatomical images were obtained using a T1-weighted MP-RAGE sequence (matrix size = 256 × 256, number of slices = 208, slices thickness = 1.0 mm, spatial resolution = 1.0 × 1.0 × 1.0 mm^3^, TE = 1.89 ms, TR = 1670 ms, field of view = 250 × 250 mm, flip angle = 9°).

Images were preprocessed using statistical parametric mapping (SPM12; http://www.fil.ion.ucl.ac.uk/spm/software/spm12/). All participants moved <3 mm during scans in in *x*, *y* or *z* planes. Data were corrected with regard to slice timing and were coregistered to the first remaining time sample. Images were coregistered to the T1-weighted image for each subject. The T1-weighted images were normalized to the standard T1 template, using non-linear transformation and the resulting transformation matrices were applied to the coregistered functional images. These normalized images were smoothed by a Gaussian kernel of 6 mm full width at half maximum. A high-pass filter (128 s) was applied on the image time series to eliminate low-frequency signals.

### Statistical analysis

#### 

***Behavioral analysis*** Statistical analyses were carried out by using SPSS 24.0 (SPSS Inc., Chigago, IL, USA). Based on the previous study reporting attentional capture effects (ACEs; capture of attention triggered by the onset of face distractor) and AHEs (extended dwelling of attention to the face distractor after attentional capture; Parks *et al.*, 2014), we also pre-defined target types as T1, T2, T3, T4 and T-baseline. Specifically, ‘T1’ refers to the target appearing at the same time as a central face distractor, and ‘T2–T4’ refer to targets occurring 1–3 s after appearance of the face distractor. ‘T-baseline’ refers to all other targets appearing without distractors. Thus, if participants’ attention were initially captured by the onset of face distractor, we would find significantly slower RT for T1 than T-baseline. Likewise, if participants’ attention were held by the face distractor after the initial capture, we would observe significantly slower RT for T2, T3 and/or T4 compared to T-baseline. Using the same paradigm, Parks *et al.* (2014) found AHEs up to T3 when face distractors were intermixed with other types of distractors, such as places (for more detail, please see Parks *et al.*, 2014). Hence, we also pre-defined target types from T1 to T4 and made other types of targets as T-baseline to detect possible attentional dwelling effects as well as initial capture effects. Repeated measures analysis of variance (rmANOVA) with three factors [emotion (neutral *vs* angry), time (T1, T2, T3, T4 and T-baseline) and group (controls *vs* SAD)] was conducted to test ACEs and AHEs for RT data. The Benjamini–Hochberg (BH) correction was used to correct the alpha level of multiple comparisons for post-*hoc t*-tests (Benjamini and Hochberg, 1995).

#### 

***fMRI data analysis*** Functional data were analyzed using a general linear model in SPM12 (http://www.fil.ion.ucl.ac.uk/spm/software/spm12/). For each participant, a whole brain voxel-wise analysis was conducted in which individual events were modeled as a canonical hemodynamic response. Each event type was first modeled for each participant using a fixed effects analysis. Individual event maps of distractor onset and offset time point for each distractor type (i.e. angry and neutral) were generated at the first-level analysis. Then, the resulting least squares parameter estimates of the height of the modeled hemodynamic response (i.e. angry onset, angry offset, neutral onset and neutral offset) were entered into a second-level analysis using a flexible factorial model to examine ACEs. The activation maps for the main effects and interaction were analyzed for each group [2 (emotion: angry and neutral) × 2 (time: on and off)]. ‘On’ refers to the onset of the face distractor and is the same as ‘T1’. ‘Off’ refers to a target appearing at the same time as the central distractor disappeared and means the offset of a face distractor. Activation for the comparison between trial types was determined by uncorrected *P-*values of less than 0.005 with a cluster extent threshold of 20 voxels, in order to achieve a reasonable compromise between type I and II errors as approved in previous studies (Lieberman and Cunningham, 2009; Kim and Giovanello, 2011; Hur *et al.*, 2016). For the analysis of small volume correction (SVC), a corrected *P* < 0.05 after family-wise error (FWE) correction was applied within *a priori* regions of interest (i.e. the attention and affective networks). Specifically, the SVC regions for distractor-processing areas (e.g. amygdala and FFA) were defined as 4 mm spheres surrounding the maximum activity identified with the localizer scan. The SVC for the insula was defined by the automated anatomical labeling template. The SVC for the IPS and TPJ was defined as cubic regions that encompassed activations labeled as right IPS and right TPJ in previous studies of attentional orienting (Vandenberghe *et al.*, 2001; Kincade *et al.*, 2005). MarsBar software (http://marsbar.sourceforge.net) and WFU Pickatlas (http://fmri.wfubmc.edu/software/pickatlas) were used to create the SVC mask regions. VOI analyses were conducted using SPM12, and beta values were extracted from all voxels within the VOI and averaged.

For AHEs, eight first-level design matrices were generated for each individual for the four time conditions (e.g. T1, T2, T3 and T4) per each distractor type to extract contrast maps for AHE. The contrast map was then generated by calculating differences between each onset condition and offset for each face distractor type. The contrast map and peak activation analysis were the same as for the ACE analyses.

## Results

### Behavioral results

Responses >150 ms or <1150 ms were excluded from all analyses. RTs during the task are reported in the Supplementary data Table 1. The rmANOVA results with three factors (i.e. group as a between-subject factor, emotion and time as within-subject factors) showed main effect of time [*F*(1.9,113.7) = 126.67, *P* = 0.01, *η^2^_p_* = 0.68] and a significant interaction effect of emotion and time [*F*(3.2,187.8) = 4.65, *P* = 0.03, *η^2^_p_* = 0.07]. To discover the origin of this two-way interaction, subsequent planned comparisons were conducted for 16 pairs with the BH correction (for similar method, please see Villemonteix *et al.*, 2017). As shown in Figure 1B, the interaction effect was driven by differential response patterns between the diagnostic groups (SAD *vs* control). Specifically, the patient group showed significantly slower RTs for T1 than T-baseline for both angry and neutral distractor conditions, indicating significant ACE by the abrupt onset of the face distractor regardless of emotion types [for angry T1, MD = 72.0 ms, *t*(30) = 11.73 and *P* < 0.001; for neutral T1, MD = 74.1 ms, *t*(30) = 9.68 and *P* < 0.001]. Further, significant AHE was found for the angry face only in the SAD group. That is, RTs for T2 with angry face distractors were significantly slower than those of T-baseline [MD = 19.6 ms, *t*(30) = 3.75, *P* = 0.001], and RTs for T3 with angry face distractor showed a trend level to significant when compared to those of T-baseline [MD = 8.9 ms, *t*(30) = 2.26, *P* = 0.031], suggesting significant attentional dwelling effects to the task-irrelevant angry face distractor in the SAD group (Figure 1B). In contrast, while the control group showed similar ACEs for both face distractors [for angry T1, MD = 59.5 ms, *t*(29) = 8.48 and *P* < 0.001; for neutral T1, MD = 64.5 ms, *t*(29) = 10.37, *P* < 0.001], no AHE was found in the control group in any type of face distractors. The only pattern we found in the planned comparison analyses were opposite patterns to the AHEs in the control group: neutral T3 [MD = −10.02 ms, *t*(29) = −2.45, *P* = 0.020] and angry T4 [MD = −9.89 ms, *t*(29) = −2.25, *P* = 0.032]. This opposite pattern to the AHEs was also found in the patient group only for neutral T4 [MD = −12.31 ms, *t*(30) = −3.12, *P* = 0.004] (Figure 1B).

### Imaging results

Imaging data from one patient and one control were excluded from further analysis due to image quality problems. See the Supplementary data for results of the functional localizer run.

#### 

***ACEs of abrupt onset of the face distractor*** Flexible factorial analyses with emotion (angry/neutral) and time (on/off) as factors in each group revealed that significant neural activations in the emotion- and attention-related regions were uniquely associated with the onset of the ‘angry’ distractor only in the SAD group. Specifically, emotion–time interaction contrast showed significant activations in the distractor- and emotion-related areas in the SAD group. The contrast of ‘Angry (On > Off) > Neutral (On > Off)’ showed significantly enhanced activity in the right amygdala, bilateral insula, left IFG and right TPJ in patients with SAD (*P* < 0.005, uncorrected, *k* > 20). The SVC analyses revealed significant interaction effects in the right amygdala and bilateral insula (*P* < 0.05, FWE-corrected, using SVC within each area) in the SAD group (Table 2). In controls, neither whole brain nor SVC analyses showed significant interaction effect in any of attention- or emotion-related regions.

**Table 2:** Brain regions showing significant interaction effects of emotion (angry > neutral) and time (on > off)^a^

**Brain region**	**Peak coordinates**			**Number of voxels**	***t***	***P***
	***x***	**y**	**z**			
**Patients with SAD**						
Right (R) amygdala/lentiform nucleus^*^	30	−12	−8	249	4.42	<0.001
R insula^*^	40	18	−1	764	4.20	<0.001
IFG	54	26	0		4.15	<0.001
Left (L) insula^*^	−40	22	−6	708	4.13	<0.001
Insula	−46	16	0		3.87	<0.001
Claustrum	−26	26	−4		3.70	<0.001
L posterior cingulate	−8	−44	22	33	3.78	<0.001
L lentiform nucleus	−26	2	−2	84	3.60	<0.001
L IFG	−58	20	16	60	3.54	<0.001
IFG	−46	20	24		3.07	0.001
R thalamus	12	−8	10	120	3.52	<0.001
Thalamus	12	−24	4		3.31	0.001
Thalamus	22	−10	14		2.66	0.005
L superior temporal gyrus	−48	−48	12	95	3.40	0.001
Superior temporal gyrus	−58	−44	20		3.04	0.002
Cingulate gyrus	−2	10	28	121	3.40	0.001
Cingulate gyrus	−4	−2	28		3.23	0.001
Cingulate gyrus	4	22	26		3.02	0.002
R inferior parietal lobule/TPJ	58	−40	28	48	3.34	0.001
Supramarginal gyrus/TPJ	64	−40	34		3.26	0.001
L caudate	−18	−6	20	22	3.28	0.001
R middle frontal gyrus	42	6	38	33	3.17	0.001
**Controls**						
R caudate	22	−38	22	61	3.92	<0.001
Cingulate gyrus	18	−34	30		3.31	0.001
R superior temporal gyrus	44	−38	8	38	3.61	<0.001
Caudate	38	−44	12		3.30	0.001
R insula	34	−40	26	33	3.01	0.002

^a^The 2 × 2 flexible factorial model analysis in each group, with a threshold of uncorrected *P* < 0.005 and more than 20 voxels. ^*^P < 0.05 after FWE correction for small volumes.

**Fig. 2:**
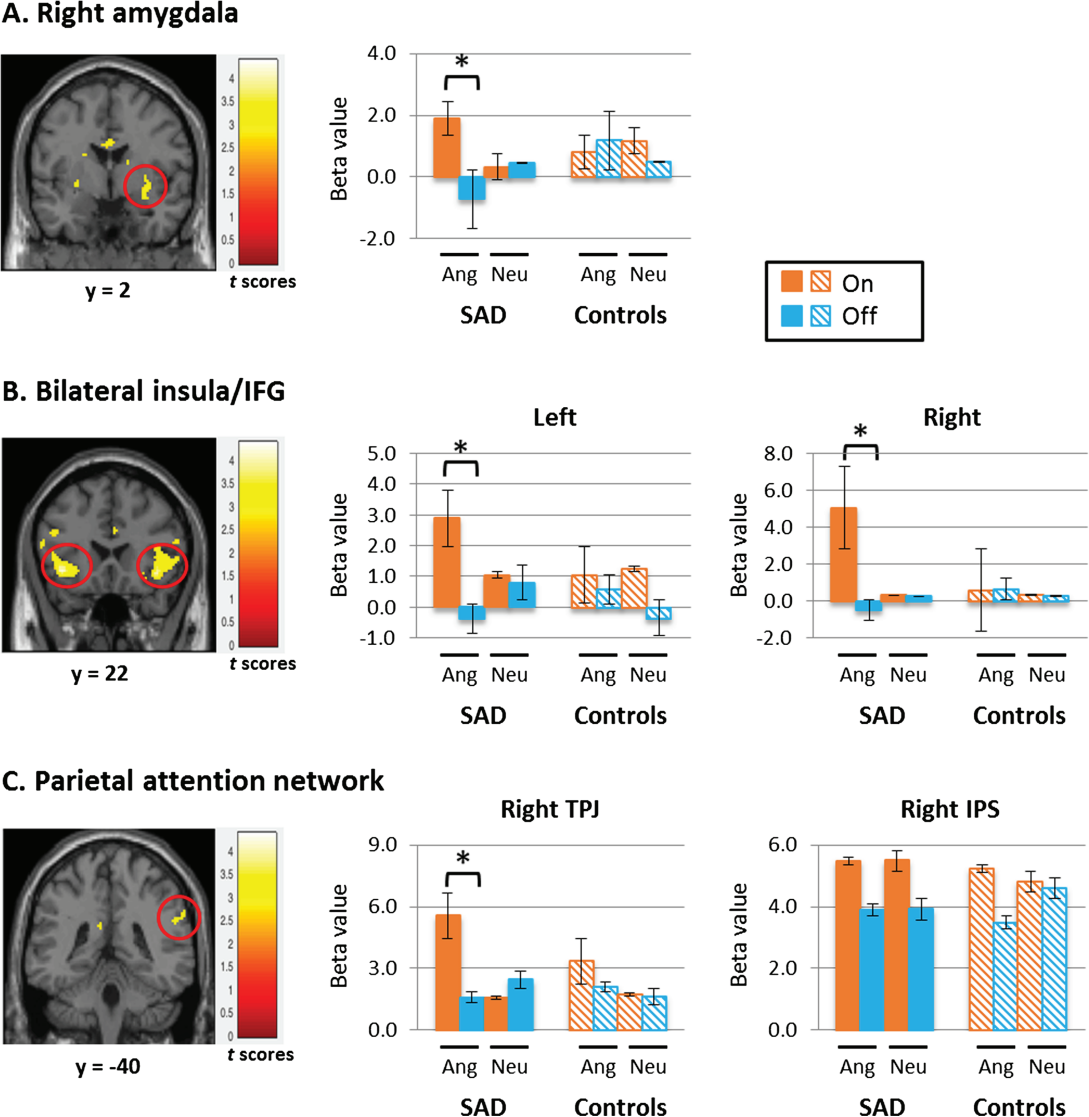
Neural responses during the initial capture of attention by face distractors in patients with SAD and healthy controls. Significant interaction effects of emotion (angry and neutral) × time (on and off) were observed in beta values of VOI in the patient group. Significant neural activation in the emotion- and attention-related regions was associated with the onset of the ‘angry’ distractor only in patients with SAD. ‘On’ refers to the onset of the face distractor and ‘off’ refers to the offset of the face distractor. Ang, angry face distractor; Neu, neutral face distractor. *P < 0.05 with paired *t*-test.

To further examine different effects of face distractors between SAD and control groups, we conducted a series of rmANOVA with three factors in emotion- and attention- related VOIs: group (control/SAD), emotion (neutral/angry) and time (on/off; Figure 2). For the analysis, we extracted the beta value from emotion- and attention-related VOIs in each group. Beta estimates of each VOI were extracted from the interaction contrast of angry (on > off) > neutral (on > off) in each participant. The rmANOVA on beta values in the right amygdala revealed a significant three-way interaction of group–emotion–time [*F*(1,57) = 15.12, *P* < 0.001, *η^2^_p_* = 0.21], an interaction between group and time [*F*(1,57) = 6.08, *P* = 0.017, *η^2^_p_* = 0.10] and a main effect of time [*F*(1,57) = 9.78, *P* = 0.003, *η^2^_p_* = 0.15). The same three-way rmANOVA on beta values in the right insula/IFG also showed a significant interaction of emotion–time–group [*F*(1,57) = 6.30, *P =* 0.015, *η^2^_p_* = 0.10], an interaction between time and group [*F*(1,57) = 6.81, *P* = 0.012, *η^2^_p_* = 0.11], an interaction between emotion and time [*F*(1,57) = 5.13, *P* = 0.027, *η^2^_p_* = 0.09] and main effects of emotion [*F*(1,57) = 4.48, *P* = 0.039, *η^2^_p_* = 0.08] and time [*F*(1,57) = 10.17, *P* = 0.002, *η^2^_p_* = 0.16]. Subsequent two-way rmANOVA on each group revealed that the significant results were led only by the SAD group, showing interaction between emotion and time [*F*(1,29) = 19.84, *P* < 0.001, *η^2^_p_* = 0.41] and main effects of emotion [*F*(1,29) = 5.19, *P* = 0.03, *η^2^_p_* = 0.15] and time [*F*(1,29) = 17.65, *P* < 0.001, *η^2^_p_* = 0.38]. A three-way rmANOVA on the left insula/IFG resulted in similar patterns of results, showing an interaction of emotion–time–group [*F*(1,57) = 9.30, *P* = 0.003, *η^2^_p_* = 0.14] and a main effect of time [*F*(1,57) = 24.81, *P* < 0.001, *η^2^_p_* = 0.30]. Post-hoc rmANOVA on each group again revealed significant effects only in the SAD group, showing an interaction between emotion and time [*F*(1,29) = 10.34, *P* = 0.003, *η^2^_p_* = 0.26] and a main effect of time [*F*(1,29) = 17.30, *P* < 0.001, *η^2^_p_* = 0.37]. Finally, results in a three-way rmANOVA revealed a significant interaction of emotion–time [*F*(1,57) = 6.84, *P* = 0.011, *η^2^_p_* = 0.10] and main effects of emotion [*F*(1,57) = 4.33, *P* = 0.042, *η^2^_p_* = 0.07] and time [*F*(1,57) = 4.41, *P* = 0.040, *η^2^_p_* = 0.07]. A three-way interaction of emotion–time–group showed marginal effects of significance [*F*(1,57) = 3.67, *P* = 0.060, *η^2^_p_* = 0.06]. Post-hoc rmANOVAs on each group confirmed significant effects only in the SAD group, showing an interaction of emotion and time [*F*(1,29) = 10.56, *P* = 0.003, *η^2^_p_* = 0.27] and main effects of emotion [*F*(1,29) = 4.16, *P* = 0.05, *η^2^_p_* = 0.13] and time [*F*(1,29) = 4.25, *P* = 0.048, *η^2^_p_* = 0.13]. All of the peak responses in above regions were significantly greater with the onset of the angry face distractor than in all other conditions in the SAD group. However, the same three-way rmANOVA on the right IPS revealed no significant emotion–time–group interaction effects. Post-hoc *t*-test results from above analyses showing significant interaction effects are presented in the Supplementary data.

#### 

**Fig. 3:**
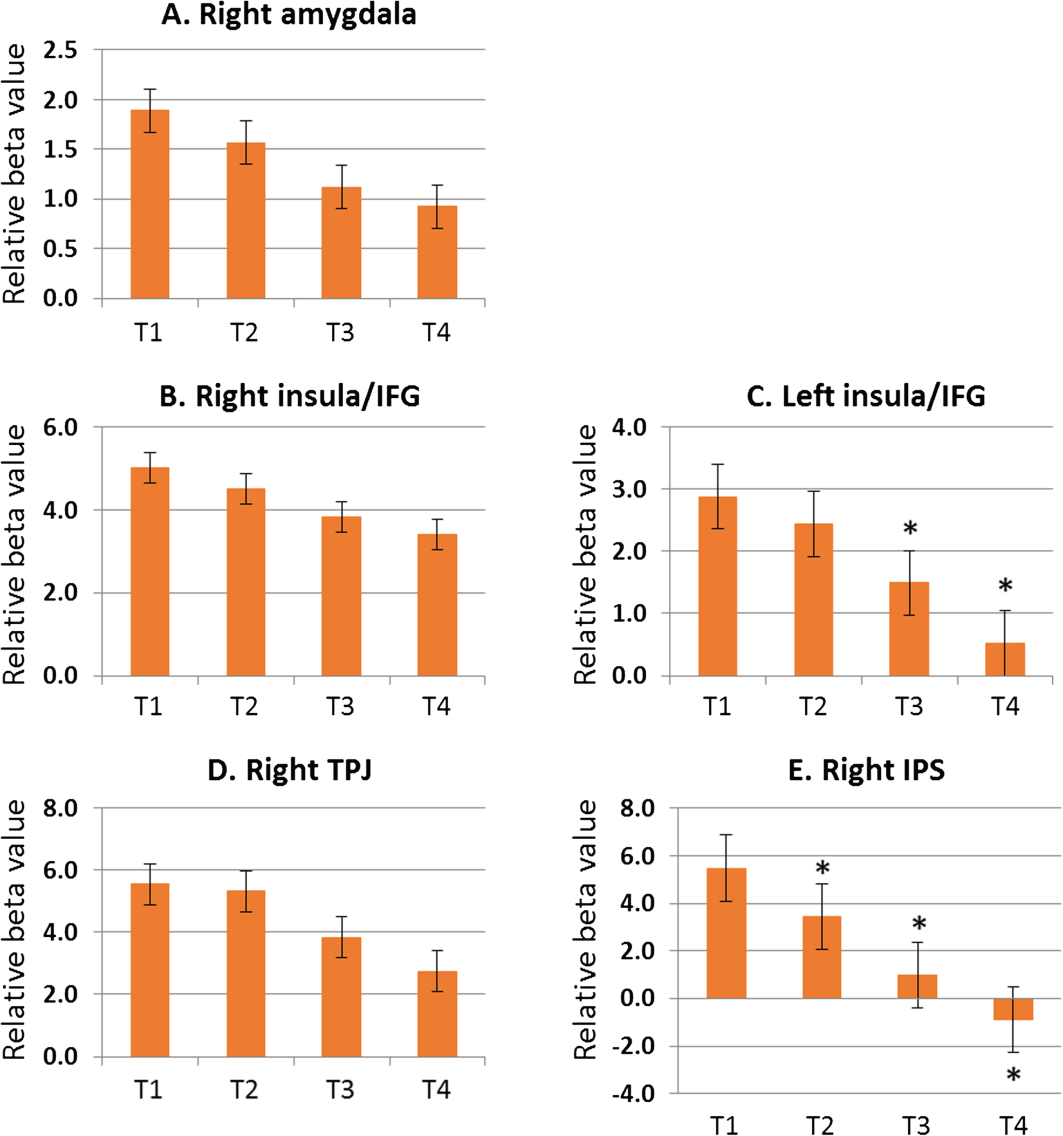
Neural responses during the holding effect of attention in patients with SAD. Relative beta value represents beta value of angry face in each of T1 to T4 relative to the off condition. Relative beta values from T1 to T4 were comparable in the right amygdala, right insula and right TPJ, suggesting that the enhanced neural activities during capture of attention in emotion- and attention-related areas were sustained for the later targets **(A, B, D)**. In addition, relative beta values from T1 to T2 were comparable in the left insula **(C)**. However, relative beta values of the IPS decreased significantly 1 s after appearance of the angry face **(E)**. *P < 0.05 with paired *t*-test compared with T1.

***Attentional holding effects*** To examine whether the behavioral AHEs of the angry face in the SAD group were reflected in neural activities on emotion- or attention-related VOIs, we extracted beta estimates of each VOI in each T1–T4 contrast compared to the off condition of the angry face. Then, we conducted a series of paired *t*-tests to examine whether the strengths of neural activity responding to each target (i.e. T1, T2, T3 and T4 of the angry face) were significantly different from baseline (i.e. off of the angry face) in each VOIs. We tested whether the enhanced neural activities related to ACE by the angry face in attention- and emotion-related areas were sustained for the later targets (i.e. AHEs) in the patient group. As shown in Figure 3, the results revealed that the strength of neural activity of the right amygdala, bilateral insula and right TPJ did not differ between T1 and T2 (all *P* > 0.05), showing AHEs by angry face distractor in the SAD group. In addition, AHEs in the right amygdala, right insula and right TPJ lasted up to the T3 and T4 targets. However, the neural activity in the right IPS decreased significantly from the T2 [MD = 2.040, *t*(29) = 4.689, *P* < 0.001] and it continued to be less at T3 [MD = 4.502, *t*(29) = 5.597, *P* < 0.001] and T4 [MD = 6.364, *t*(29) = 6.038, *P* < 0.001] relative to T1, showing the absence of AHEs.

## Discussion

The present study provides behavioral and neural evidences of extended attentional hold, as well as initial attentional capture, to the emotional distractor, which impaired performance in patients with SAD. Furthermore, the underlying neural mechanism of persistent attentional bias involved the bottom-up attention network of the TPJ and IFG, along with emotion-related regions of the amygdala and insula.

This population display vigilance for negative evaluation and difficulty ignoring a social threat and allocate attention to threats in the environment (Schultz and Heimberg, 2008). In a previous study, subsequent holding of attention after the initial involuntary capture of attention to distractors was dependent upon the ongoing distractor context in healthy individuals (Parks *et al.*, 2014). When the task-irrelevant distractor was given as angry or neutral faces with neutral places, the faces specifically held the captured attention (attentional bias was not persistent for neutral places). On the other hand, when only face stimuli (e.g. angry and neutral) were presented as distractors without other types of stimuli (i.e. ‘the face-distractor context’), the face no longer held the attention. That is, when salient stimuli were presented in succession, the stimuli no longer had salience. However, the present study showed that the angry face has marked salience over the neutral face in patients with SAD unlike healthy individuals; the angry face held captured attention even when it came with the neutral face as a distractor in the present study. This is in line with a previous finding that training disengagement from a threat reduces behavioral indices of social anxiety, indicating that the difficulty in disengaging attention from a threat is a critical process in maintenance of SAD (Heeren *et al.*, 2011). Attentional bias modification training to disengage from a threat and to attend to positive stimuli (Heeren *et al.*, 2012) would be essential to reduce symptoms of social anxiety in a patient’s everyday life.

From the imaging results not shown here, we found that task-irrelevant face distractors affected activity in the visual processing areas regardless of emotional condition in both groups. The appearance of the face distractor enhanced activities in face-processing regions and reduced activities in target-processing regions at the same time, and the opposite occurred when the face distractor disappeared in accordance with our previous study (Kim and Hopfinger, 2010). These findings suggest that both groups have comparable visual information processing no matter which emotion was on the face. The same pattern was exhibited in the IPS; both groups showed similar activity patterns (Figure 2C). Although right IPS activity changed slightly depending on the onset and offset of distractors, it was engaged consistently throughout the task regardless of the emotional condition in both groups. Additionally, this region showed no holding effect after the appearance of the negative face distractor (Figure 3E), which agree with the previous finding of specific impairment in the orienting network but preserved function in the executive network of patients with SAD (Heeren *et al.*, 2015a, b). Because the IPS is mainly involved in goal-directed (top-down) control of attention (Bundesen, 1990; Corbetta *et al.*, 2008), the present findings suggest that the dorsal attention network is relatively intact in patients with SAD.

On the other hand, the TPJ and IFG, which are responsible for the stimulus-driven (bottom-up) attentional control mechanism, are specialized in detecting behaviorally relevant stimuli, particularly when they are salient or unexpected (Corbetta *et al.*, 2000; Corbetta and Shulman, 2002). Increased functioning of this ventral attention networks was proposed in anxiety disorders (Sylvester *et al.*, 2012). The present findings show that this ventral attention network is congruent with the emotion-related regions in response to a social threat in patients with SAD. Controls engaged these regions consistently regardless of the emotional condition and the onset or offset of distractors, whereas patients with SAD showed heightened activity in response to the angry face distractor and reduced activity when the angry distractor disappeared (Figure 2). In a healthy state, the ventral attentional network should be suppressed when attention is focused to prevent reorienting to distracting events (Corbetta *et al.*, 2008). The neural mechanism underlying attentional bias to social threats in patients with SAD seems to be in the hyper-reactivity of the bottom-up attentional network, along with an excessively responsive affective system. The IFG, furthermore, has been found to be involved in the persistence of cognitive processing of social exclusion in patients with SAD (Heeren *et al.*, 2017). The amygdala is surely a key structure involved in the rapid and unconscious processing of emotional faces (Etkin and Wager, 2007). Previous studies reported that amygdala reactivity to facial emotions appears to be a determinant of automatic negative evaluative response tendencies; with regard to social anxiety, amygdala hyper-reactivity makes a contribution to negative cognitive biases (Dannlowski *et al.*, 2007; Goldin *et al.*, 2009). The amygdala is involved not only in automatic attention processing but also in cognitive attention processing of threatening stimuli (LeDoux, 1995). Importantly, our results also show an extended dwelling effect of biased attention at the neural level (Figure 3). The long-lasting interruption of ongoing selection in the top-down control, resulting in a shift of attention toward a threat, would play a role in provoking and maintaining social anxiety in patients with SAD.

There are several limitations to our study. First, one could question that basic cognitive abilities or the attentional span may be responsible for different patterns of attentional bias found between the SAD and control groups in this study. Although we did not measure basic cognitive functions or attention spans in our participants, the educational levels were not significantly different between the two groups. Second, our fMRI design is not completely perfect to examine AHEs due to the nature of our continuous performance task. That is, the targets appeared every second, thus it was not eligible to directly contrast T1 *vs* T2 events to examine possible holding effects. In our task, we jittered event timing by varying the distractor presence and absence durations based on a previous fMRI study using the same continuous performance task (Kim and Hopfinger, 2010). To measure AHEs, we thus compared results from the ‘T1 > Baseline contrast’ to results from the ‘T2/T3/T4 > Baseline contrast’ in each brain areas of interest. As results, we found that the AHEs were differently presented depending on brain areas. That is, we demonstrated significant AHEs in the right amygdala, bilateral insula and right TPJ, whereas the effect was absent in the right IPS. If the effects of T1 affected the subsequent target events (T2–T4), we would have expected to see the same results of AHEs in all the brain areas. Similarly, none of AHEs were significant in the control group in any of the brain regions. Thus, despite successive nature of our target events, our jittering method allowed to examine possible AHEs in our participants. Third, in our task, we only included negative emotional faces along with neutral faces without using positive emotion faces (e.g. happy faces). Thus, the persistent attentional bias effects to the negative emotion faces might be resulted from the emotion in nature regardless of its negativity or valence. Further investigation including both happy and angry faces or both positive and negative emotional stimuli other than faces could elucidate the nature of attentional bias to a threat in SAD. Lastly, there could be possible confounding effects of depression and/or serotonergic medication, as BDI scores and medication status were different between groups. There included four patients who have comorbid depressive disorders. Clinically, SAD and depressive symptoms often covary (Heeren *et al.*, 2018). Thus, we included these participants when depressive disorders were judged to have been accompanied by SAD. Further research with a larger population including mood disorders is needed to exclude these confounding effects.

In summary, the present study demonstrates that both attentional mechanisms, namely initial capture of attention and the subsequent holding effect of biased attention to a social threat, underlie the pathophysiology of SAD. Inefficient stimulus-driven control to task-irrelevant social threats in the ventral attentional network, such as the TPJ and IFG, along with the amygdala hyper-reactivity, seems to underlie the attentional bias in patients with SAD. Further research with a larger sample size would help elucidate the neural-behavioral relationships on attentional bias, which can be used as a tool to measure psychopathology or predict treatment responses.

## Funding

This work was supported by the Basic Science Research Program through the National Research Foundation of Korea funded by the Ministry of Education (2017R1D1A1B03036385) and the Ministry of Science, ICT and Future Planning (2014R1A1A1004553).

## 

*Conflict of interest.* None declared.

## Supplementary Material

Supplementary Data
