# Corrigendum to: Neural evidence for persistent attentional bias to threats in patients with social anxiety disorder

**DOI:** 10.1093/scan/nsy115

**Published:** 2018-12-19

**Authors:** So-Yeon Kim, Jung Eun Shin, Yoonji Irene Lee, Haena Kim, Hang Joon Jo, Soo-Hee Choi

**Affiliations:** 1 Department of Psychology, Duksung Women’s University, Seoul, Republic of Korea; 2 Department of Psychiatry, Seoul National University Hospital, Seoul, Republic of Korea; 3 Department of Psychological and Brain Sciences, Texas A&M University, USA; 4 Department of Neurology; 5 Department of Neurosurgery, Mayo Clinic, Rochester, MN, USA; 6 Department of Psychiatry, SNU College of Medicine, Institute of Human Behavioral Medicine, Medical Research Center, Seoul National University, Seoul, Republic of Korea

Soc Cogn Affect Neurosci (2018), 1327.

DOI: https://doi.org/10.1093/scan/nsy101

Footnotes indicating that the first two authors contributed equally have been added to the article.

