# Supplementary material for: Neural evidence for persistent attentional bias to threats in patients with social anxiety disorder
Source: Soc Cogn Affect Neurosci. 2018 Nov 15;13(12):1327–36. doi: 10.1093/scan/nsy101 (PMC6277744; doi:10.1093/scan/nsy101)
Supplement: Supplementary Data [file nsy101_supp.docx]

**Supplementary material**

**Supplementary methods**

**Functional localizer run**

Participants were asked to focus on a green square dot at the center at all times. To localize brain areas for target processing, a checker-board flashed in the upper right visual field (i.e., a target location) in 1/3 of the trials in a localizer scan. Each square of the checkerboard reversed contrast (black/white) at a frequency of 4 Hz. To localize brain areas for distractor processing, a neural or angry face distractor was presented in the center in another 2/3 of the trials in the scan. The sequence of the run was: (1) peripheral checkerboard, (2) central angry face, and (3) central neutral face, appearing for 10 seconds, with 10 seconds of fixation between conditions. The sequence was repeated for eight times.

**Supplementary results**

**Imaging results**

*Functional localizer run.* First, we identified distractor- and target-related regions using data from the localizer scan in the control group (*p* < .05, FWE-corrected for whole brain). The analysis identified two regions of distractor-related areas, namely the bilateral amygdala (left, x, y, z: −28, 4, −10; right, x, y, z: 22, −4, −14) and left fungiform gyrus (x, y, z: −18, −92, −8), showing significantly higher activity for the angry face than the target stimulus. Target-related areas were also identified in the left lingual gyrus (x, y, z: −8, −80, −4) using the contrast of peripheral target stimulus > distractor of neutral face.

*Attentional Capture Effects (ACEs)* *of abrupt onset of face distractor.* Specifically, the peak response in the right amygdala was significantly greater with the onset of angry face distractor compared to all other conditions in the SAD group: (1) greater than the offset of the angry face [MD = 2.601, *t*(29) = 4.711, *P* < .001]; (2) greater than the onset of the neutral face [MD = 1.571, *t*(29) = 2.603, *P* = .014]; (3) greater than the offset of the neutral face [MD = 1.429, *t*(29) = 2.824, *P* = .004]. Further, the peak responses in the bilateral insula were significantly greater for the onset of angry face compared to all other conditions in patients: (1) greater than angry face-off [left, MD = 3.255, *t*(29) = 5.401, *P* < .001; right, MD = 5.509, *t*(29) = 5.936, *P* < .001]; (2) greater than neutral face-on [left, MD = 1.838, *t*(29) = 2.640, *P* = .007; right, MD = 4.702, *t*(29) = 5.544, *P* < .001]; (3) greater than neutral face-off [left, MD = 2.086, *t*(29) = 3.699, *P* = .001; right, MD = 4.781, *t*(29) = 4.110, *P* < .001]. The activation of the right TPJ was significantly greater for angry face-on compared to all other conditions: (1) greater than angry face-off [MD = 3.965, *t*(29) = 3.244, *P* = .003]; (2) greater than neutral face-on[MD = 3.987, *t*(29) = 3.297, *P* = .003]; (3) greater than neutral face-off [MD = 3.112, *t*(29) = 2.709, *P* = .011]. Finally, the same ANOVA revealed no significant interaction effects in the right IPS VOI in the SAD group.

**Supplementary Table 1.** Reaction times (ms) on the continuous performance task

| **Group** | **Emotion** | **T1 (On)** | | **T2** | | **T3** | | **T4** | | **T (Base)** | |
| --- | --- | --- | --- | --- | --- | --- | --- | --- | --- | --- | --- |
|  |  | Mean | SD | Mean | SD | Mean | SD | Mean | SD | Mean | SD |
| Controls  (*N* = 30) | Neutral | 637.1 | 66.0 | 567.9 | 52.6 | 562.6 | 57.8 | 562.7 | 56.6 | 572.6 | 60.0 |
|  | Angry | 632.8 | 74.9 | 575.7 | 64.1 | 568.3 | 64.2 | 563.5 | 66.9 | 573.4 | 62.2 |
| SAD  (*N* = 31) | Neutral | 669.7 | 72.3 | 600.9 | 56.8 | 588.0 | 61.5 | 583.2 | 57.5 | 595.5 | 62.9 |
|  | Angry | 662.7 | 71.8 | 610.3 | 61.3 | 599.5 | 61.4 | 593.0 | 61.6 | 590.7 | 63.1 |

SAD, social anxiety disorder.
